# Intervention strategies for type 2 diabetes prevention in high-income countries targeting low socioeconomic groups: a scoping review

**DOI:** 10.3389/fpubh.2025.1583817

**Published:** 2025-07-25

**Authors:** J. Thylefors, M. Annersten Gershater, E. Mangrio, S. Zdravkovic

**Affiliations:** Faculty of Health and Society, Malmö University, Malmö, Sweden

**Keywords:** intervention studies, low socioeconomic status, prediabetes, prevention, type 2 diabetes

## Abstract

**Introduction:**

Type 2 diabetes is increasing worldwide, and the trend is also observed in Sweden. In Malmö, the third largest city in Sweden, the prevalence has doubled. Populations with lower socioeconomic status have a higher prevalence and poorer outcomes, making preventive interventions targeting these groups increasingly important.

**Objective:**

To investigate the types of interventions that have been tested and reported regarding the prevention of type 2 diabetes targeting low socioeconomic populations and are applicable in a high-income country.

**Methods:**

Based on a systematic search strategy developed using the People, Concept, and Context model, the databases CINAHL, PubMed, and Web of Science were searched in January 2024 and updated in December 2024, and EMBASE was searched in May 2025. A flowchart of the screening process has been created. From the selected studies, data were extracted, charted, and the findings were compiled in a narrative form.

**Results:**

Seventeen studies were included, 12 were conducted in the United States and five in Europe. Most used culturally adapted diabetes prevention programs, and a higher proportion of participants were women. Key features included flexibility in attendance and format, development through a community-based participatory approach, gender-specific groups, and the involvement of significant others. Increases of physical activity proved challenging within broader lifestyle interventions. Screening interventions were conducted in community and healthcare facility settings, as well as through a school-and community-based program. Challenges with enrollment and retention were commonly reported.

**Conclusion:**

There is a need for more interventions in the European context and for interventions to engage more men with strategies such as male peer coaches and community screening in locations frequented by men. Longer time frames and sustained engagement strategies are necessary to reach and retain groups with low socioeconomic status in preventive type 2 diabetes interventions.

## 1 Introduction

Type 2 diabetes (T2D) is increasing worldwide (1). This trend is primarily driven by the risk factors of overweight and obesity, while non-modifiable factors include age, genetic predisposition, and population-specific genetic susceptibility (2). In Malmö, one of the three largest cities in Sweden, characterized by a demographically young population with a wide range of countries of origins, T2D prevalence has doubled between 2011 and 2018 (3).

In addition to its impact on individuals’ health-related quality of life (4), T2D is associated with an increased risk of mortality (5). However, the disease shows a heterogenous pattern. For example, the excess risk of overall-cause mortality among patients with T2D has generally declined in Sweden over recent decades, but to varying extents across subgroups of T2D patients (5, 6).

Socioeconomic status (SES) affects health and is commonly measured using the indicators educational level, occupation, and income, which are intercorrelated but not interchangeable (7). Additionally, individual and neighborhood socioeconomic factors might interact (8). An international clinical tool to measure SES is not available (9). Regardless of whether the context is a low-, middle-, or high-income country, T2D tends to affect individuals with lower SES to a greater extent (10–12). Furthermore, people with low SES are often affected by the disease in a more detrimental manner compared to those with higher SES (11, 13). These inequalities between socioeconomic groups persist in high-income countries with predominantly publicly funded healthcare and education systems, such as those in Scandinavian countries (10, 14).

Early detection of prediabetic states or T2D is important for improving outcomes. Early detection may initially be guided by non-invasive screening (15). Diagnosis will be based on the biomarkers HbA1c, fasting blood glucose and/or oral glucose tolerance test (OGTT), with cut-off points that vary according to different guidelines (16, 17). As the disease often evolves without any prominent symptoms over several years, undetected cases are common, and complications may already be present by the time the condition is finally detected (16–18). In high-income countries, it is estimated that 29% of all diabetes cases are undiagnosed, compared to 45% globally (18). If identified at an early stage and significant weight loss is achieved, remission of the disease may be possible for some individuals (19).

Lifestyle interventions have been shown to be effective in preventing T2D in subjects with dysglycaemia (20–24). To implement these interventions on a larger scale, various structured programs have been developed internationally. National diabetes prevention programs (NDPPs) refer to nationwide initiatives designed to systematically provide education and support to at-risk individuals, especially in the areas of healthy eating, physical activity, and weight loss. Local or regional initiatives, which can be run by health organizations, clinics, or community groups are referred to as diabetes prevention programs (DPPs). Examples of NDPPs exist in countries such as Finland, the United States, and England (21, 25, 26). In Sweden, prevention is the responsibility of primary care, which is organized regionally (27), and there is no NDPP available in the country.

It has been reported that T2D interventions do not sufficiently reach those with a lower educational background, and that completers of lifestyle interventions may have a higher educational level than non-completers (28, 29). This highlights the need for the development of different strategies to reach these populations. Interventions aiming to prevent T2D should be adapted to the specific needs and challenges of different populations, as a one-size-fits-all approach is considered likely to fail in reversing the trend of T2D prevalence (2, 9, 17). Therefore, we aimed to investigate the types of interventions that have been tested and reported regarding the prevention of T2D, targeting populations with lower SES and are applicable in a high-income country. The results will inform future interventions in the city of Malmö.

## 2 Materials and methods

### 2.1 Study design

This scoping review was conducted following the framework of the Joanna Briggs Institute (30, 31). Furthermore, recommendations were followed regarding the enhancement of scoping study methodology through consultation with stakeholders (32). The purpose of the consultation was to assess the perceived priority of the identified interventions within the local Malmö context. To this end, we presented preliminary findings to stakeholders, including the chair and a member living with T2D from a local Diabetes Association in southwest Sweden. The stakeholders were asked to identify which intervention they considered most important to initiate in Malmö. Their perspectives were documented in writing during a two-hour, in-person meeting and have been incorporated into the discussion section.

An *a priori* protocol was developed for the scoping review (33), which was followed with the exception that policymakers were not included as stakeholders, as we, at this stage, are aiming to inform future research-driven interventions in Malmö.

The methods and results are reported according to the Preferred Reporting Items for Systematic Reviews and Meta-Analyses Extension for Scoping Reviews (PRISMA-ScR) Checklist (34). As the International Prospective Register of Systematic Reviews (PROSPERO) does not register scoping reviews, this scoping review was not registered.

A critical appraisal of individual studies was not conducted, in line with established guidance for scoping reviews (31), as the aim was to map the existing intervention strategies rather than to synthetize findings based on methodological rigor.

The research question was as follows: What types of interventions have been tested and reported regarding the prevention of T2D targeting adult populations with lower SES, which are also applicable in high-income countries? Studies applicable to a high-income context were defined as those conducted in high-income countries.

### 2.2 Eligibility criteria

Eligibility criteria is additionally shown in the Population, Concept, Context mnemonic, which is presented in the published protocol (33).

Intervention studies addressing adult populations aged over 18 years with low SES were included. As there is no international clinical tool to assess SES, low SES as defined by the authors of the articles was accepted. The risk for T2D had to be stated in studies eligible for inclusion and tested according to the standard biochemical methods (fasting blood glucose, HbA1c, and/or oral glucose tolerance test), and/or estimated by a questionnaire tool for T2D prediction, and/or identified as being at higher risk due to ethnic predisposition and being overweight or obese, or having a history of or risk for gestational diabetes (GD). The interventions for prevention and screening had to be conducted in high-income countries, defined and listed in the World Bank classification (35).

Reviews and studies with a design other than a quantitative design were excluded. Protocols for planned studies and grey literature were not searched as we aimed to map tested and reported interventions. In addition, we aimed for the search strategy to be systematic, transparent and reproducible, which cannot be ensured regarding grey literature (36). Studies conducted in low-or middle-income countries were excluded as their transferability to a high-income country setting was deemed limited. Studies published in a language other than English were excluded. The year of publication was not restricted in the searches of CINAHL, PubMed, and Web of Science, but was limited to 2020–2025 in EMBASE.

### 2.3 Data collection

**Figure 1:**
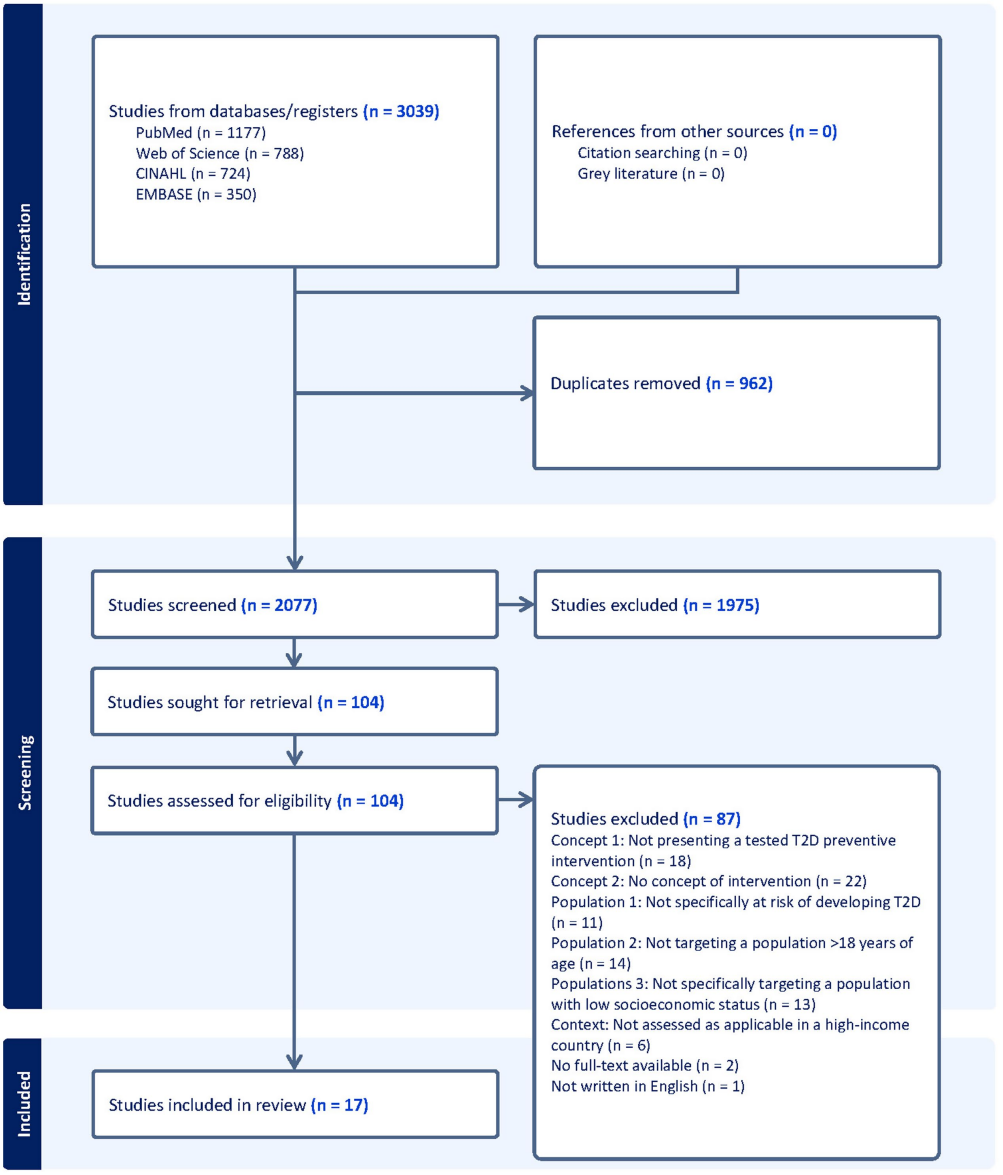
PRISMA flow diagram intervention strategies for type 2 diabetes prevention in high-income countries targeting low socioeconomic group: a scoping review.

The search strategy (Supplementary Table 1) was developed in collaboration with research librarians at Malmö University and followed the Peer Review of Electronic Search Strategies (PRESS) guideline (37). The search strategy aimed to systematically locate published studies. Searches were conducted in January 2024 and were checked for updates in December 2024. The databases searched were CINAHL, PubMed, and Web of Science. Additional searches were performed in EMBASE in May 2025 to verify that no studies meeting the inclusion criteria had been missed. Following the search, all identified citations were collated and uploaded into Covidence (38), where duplicates were removed. Titles and abstracts were then screened independently by two reviewers from the research team (JT screened all of them, and MAG, EM, and SZ each screened one third; JT alone screened all titles and abstracts in EMBASE) for relevance to this review. Subsequently, the full texts of selected citations were assessed in detail independently by the reviewers in the same manner as mentioned above, for relevance. Any doubts or disagreements were resolved through discussion within the research team at all stages of the review process. Reasons for exclusion of studies at the full-text stage, for not meeting the inclusion criteria, were recorded. The reference lists of all included studies were manually cross-checked for additional studies, and no additional studies were found to be relevant. The results of the search and the study inclusion process are presented in the PRISMA flow diagram (Figure 1).

### 2.4 Data analysis

A data extraction form was developed for this scoping review (Supplementary Tables 2, 3). The form included information on study characteristics (first author, country, year of publication, and title), study aim, design, and setting. Participant-related data included sample size, mean age, gender distribution, and risk group for T2D (prediabetes, a history of or risk for gestational diabetes, ethnicity, or obesity). Definitions of low SES, as provided by the study authors, were also recorded. Furthermore, the form covered details on the intervention content, duration, follow-up time, and a conclusion of results and comments.

Data extraction was initially carried out by two reviewers independently, JT reviewed all the included studies for extraction and MAG, EM, and SZ extracted data from two studies each. Doubts and disagreements were resolved through discussion within the research team. A consensus was achieved after extracting six studies, and thereafter, JT performed data extraction for the rest of the included studies. All research team members validated the narrative synthesis.

## 3 Results

Figure 1 illustrates the PRISMA flow diagram of the study inclusion process. From our search, the articles (*n* = 3,039) were uploaded into Covidence (38). Duplicates, (*n* = 962), were removed and 2077 titles and abstracts were screened. In total, 104 articles were assessed in full text, of which 17 articles met the inclusion criteria.

The data extracted from the included studies are presented in Supplementary Table 2 (lifestyle interventions) and Supplementary Table 3 (screening interventions). The included intervention studies were published between 2008 and 2023. The studies were conducted in the United States (*n* = 12); one in a multinational collaboration within the European Union (EU) involving Belgium, Bulgaria, Finland, Greece, Hungary, and Spain; one in England; one in Finland; one in France; and one in Sweden. The studies had heterogeneous designs, including various methods of comparative and observational studies, which are specified in the Data Extraction form (Supplementary Tables 2, 3).

Among the studies where the location of the intervention was specifically stated, the majority reported an urban context (39–46), while others reported a diverse geographical setting (47), a rural setting (48), a digital setting (49), a suburban setting (50), a county setting (51), and a school-and community-based setting (52). Two studies were from a primary care setting (42, 53).

Interventions were conducted in collaboration with non-governmental organizations in some studies from the United States (39–41, 47), and with an advocacy group and an enterprise in a large-scale screening intervention study from Europe (52).

The majority of the interventions were based on education and support for lifestyle behavior change (39–41, 43–49, 51, 53–55), and three of the studies were screening interventions aimed at detecting dysglycaemia and T2D (42, 50, 52). Ten of the intervention studies involved adaptations based on principles derived from existing NDPPs/DPPs for groups with low SES or that investigated this group’s preferences for program format, one of which was from Finland (53), and the others were from the United States (39–41, 43, 44, 46, 49, 51, 55). One study tested an education program specifically developed for a deprived population in Paris (45). Another study evaluated a community-based participatory approach for adapting a diabetes empowerment education program for an immigrant population in South Texas (48). In one study conducted in California, a food bank-delivered intervention was tested on a food-insecure population, consisting of monthly distributions of diabetes-appropriate food packages, text-based health education, and referrals to health care (47). Another study from the United States tested a mindfulness training intervention on distress, weight gain and glucose control for low-income pregnant women with obesity (54). Three European studies focused on screening interventions targeting groups with low SES, one of which was conducted in England, one in a multinational collaboration with Belgium, Bulgaria, Finland, Greece, Hungary, and Spain, and one in Sweden (42, 50, 52).

### 3.1 Outcome measures

The outcome measures in the included studies varied widely depending on the objectives, but most frequently included clinical indicators, anthropometric data, self-reported measures, and/or processes measures, see Supplementary Table 4.

### 3.2 Risk for T2D

Risk group for T2D was defined in various ways in the articles: having prediabetes and obesity, self-reported or high scores on risk tests, obesity and being pregnant, population expected to have a high prevalence of undiagnosed diabetes, ethnic group with a genetic predisposition prone to develop T2D, a general population with an extraordinarily high mean body mass index, ethnic group with a genetic predisposition prone to develop T2D and a history of GD in the past 3 years, body mass index > 25 kg/m^2^ or 23 kg/m^2^ for individuals of Asian ancestry in addition to having prediabetes, and past GD or a positive score on a risk questionnaire.

The duration of the interventions aimed at preventing T2D by influencing lifestyle behavior varied between 8 weeks and 12 months, with follow-up times ranging from at the end of an intervention to 24 months after the intervention was completed.

### 3.3 Demographics

Low SES was defined in various ways in the included articles: as low income, low income or socioeconomic deprivation assessed in relation to federal poverty level or according to an index score, enrolled in safety-net insurance plans, socioeconomically deprived, from an area with low socioeconomic profile, or low education and high unemployment rates. SES indicators used in the included studies are presented in Supplementary Table 5.

The participants in studies presenting T2D prevention interventions based on education and/or support for healthy lifestyle had a mean age ranging from 36 to 55.6 years (39–41, 43–49, 53). In the studies addressing women due to history of or risk for GD, the mean age ranged from 26.9 to 31.9 years (51, 54, 55). In the studies focusing on screening interventions, the median age of participants was 50 (50), respectively mean of 41.1 years (52), and in one of the studies, age was categorized in decades, with the majority of participants being 40–59 years old, but stretched from < 40 years old to 80 + years old (42).

### 3.4 Key intervention features

Key intervention features are additionally presented in Supplementary Table 6.

#### 3.4.1 Cultural adaptations

Six studies primarily or exclusively focused on a defined ethnic group known to have a higher risk of T2D (43, 44, 46, 48, 51, 55), of which all were conducted in the United States. In seven studies, it was reported that the intervention was offered in a language spoken by the participants (44, 46–49, 51, 55). Cultural adaptation was reported as a key component in some of the studies (44, 48, 51); for example, in one intervention, discussions were conducted during group sessions about culturally driven beliefs about diabetes (51). In the digital DPP adapted for a low income population study, bilingual and bicultural health coaches were present (49).

#### 3.4.2 Gender focus

Four studies targeted a specific gender in their interventions. Three of these studies focused on women with a history of or at risk for GD (51, 54, 55), and one study aimed to engage men of color with prediabetes in an adapted NDPP, led by male lifestyle health coaches (46). Overall, there were more female than male participants in the included intervention studies, with 16,999 women compared to 11,406 men. When considering only the interventions aiming at influencing healthy lifestyle (and excluding the screening interventions), there were 7,872 female participants compared to 3,728 male participants in the included studies.

#### 3.4.3 Intervention formats

The interventions aimed at educating and supporting lifestyle behavior change were offered in different formats. Most were primarily delivered in group settings (39–41, 43–46, 48, 49, 51, 54, 55), including one that was digital (49), and were conducted in the United States and Paris. One California study provided text-based health education along with diabetes-appropriate food individually to participants (47). Another study, conducted in Finland, was based on giving participants an option to, together with health professionals, choose group-based or individual counseling. The majority of individuals in the study preferred individual rather than group-based intervention, and both intervention types were equally effective, with only a few exceptions (53).

#### 3.4.4 Participant-based and culturally adapted with inclusion of significant others

Among the studies reporting significant impact on lifestyle changes and/or weight loss and/or blood glucose levels due to the intervention, one example is from the United States, focusing on peer-led workshops with low literacy curriculum developed by and for their own community partly based on principles derived from DPP. In this intervention, participants were permitted to bring a family member, friend, or caregiver to the sessions. The peer leader pairs shared similar socioeconomic backgrounds and health issues as the participants. Sixty-four per cent of participants completed at least half of the eight 90-min peer-led sessions at community sites, and the intervention also offered a buddy system for contact between meetings (43). Significant others were also invited to the sessions in another study from the United States: It was a community-based, culturally tailored, literacy-sensitive adaptation of the DPP, which reported a one-year retention rate of 94% (44). In one study, also from the United States, focusing on the community-based participatory adaptation of a diabetes empowerment education program, childcare was provided at the same locations as the meetings, and in the intervention group, 79.2% of participants attended at least half of the eight meetings (48). Similar to the previously mentioned study, childcare was provided, as the community-based participatory process identified it as a means to enhance attendance at the sessions in an adapted DPP for women with a history of GD. In the study, 89.5% of participants completed at least four of the eight sessions (51).

#### 3.4.5 Attendance, completion, and retention challenges

A large proportion of participants not completing the intervention, participants attending fewer than half of the sessions, difficulties with enrollment, and/or low adherence to accelerometer use in the intervention were some aspects highlighted in the studies (39–41, 45, 51). For example, one study from the United States found that low completion or attendance rates were due to life circumstances, job insecurity, competing life issues, and logistical barriers (41). In the study conducted in Paris, the workshops were planned together with the participants to suit their availability. Nonetheless, issues with the retention rate were observed, and it was discussed whether a family approach and language support could have helped limit participant attrition (45).

One study from the United States offered a catch-up option for participants who missed a session, allowing them to make up the session via telephone after receiving the materials by email or post (46). Coaches also reached out to participants between sessions and offered make-ups as needed in a study testing the NDPP on maternal health in a low-income group in the United States (55).

#### 3.4.6 Challenges of changing physical activity

In one study on changes in physical activity due to an intervention based on the DPP curriculum targeting economically disadvantaged adults with prediabetes in an urban context in the United States, the outcome was described as “disappointing” (40). Participants did lose weight, but they did not significantly change their physical activity levels. This was discussed as possibly being linked to the outcome that, as changes in their diet alone were so effective, the participants may have had less interest in becoming physically active. The study also suggested future research into qualitative work examining the lived experiences of individuals facing multiple barriers to physical activity (40). In another study from the United States, it was similarly concluded that meaningful improvement in physical activity was not achieved, although modest weight reduction and a reduction in HbA1c were observed as a result of the intervention (44). This was discussed as possibly being related to a lack of emphasis on this aspect of the intervention, as well as participants living in neighborhoods not conducive to physical activity (44).

#### 3.4.7 Screening interventions

Three of the included studies addressed screening of prediabetes and T2D (42, 50, 52). Two of these approached screening by initially administering the Finnish Diabetes Risk Score questionnaire and then conducting invasive testing on those with elevated scores (50, 52). Another study invited participants identified through the general practice clinical information system as older than 40 years and with a body mass index greater than 25 kg/m^2^. In the latter study, conducted in deprived areas in England, a low diagnosis rate of 1 in 70 screened participants was found, and practical obstacles were also reported, for example, difficulties in identifying target subjects through the information system. However, the practice staff deemed that the pilot study was successful because it provided opportunities to raise awareness about diabetes and offer advice on weight loss, diet, and other lifestyle changes.

In one of the screening interventions, community-versus facility-based screening was evaluated (50). Venues for community-based screening included shopping malls, local organizations, associations, women’s centers, and swimming facilities, where screening was offered at various times throughout the day, every day of the week. The results showed that community-based screening reached more individuals born in Africa and Asia, while facility-based screening reached more people born in Sweden and other European countries. The screening intervention was found to reach more women than men, regardless of whether it was conducted at community sites or at facilities.

In a large-scale study, the Finnish Diabetes Risk Score questionnaire was assessed for screening undiagnosed T2D and dysglycaemia among vulnerable populations across Europe, through self-administrated Finnish Diabetes Risk Score questionnaires that were collected from parents of primary-school children. High-risk families were identified if at least one parent fulfilled the country-specific cut-off point for Finnish Diabetes Risk Score. They were then invited to take part in measurements of weight, height, waistline circumference, blood pressure and blood samples were drawn of fasting plasma glucose, serum total, high-density lipoprotein cholesterol and triglyceride levels, which were carried out in local community centers or during home visits. In the study, it was concluded and discussed that Finnish Diabetes Risk Score can be applied for a cost-effective and practical first-level screening of undiagnosed T2D, with a most suitable cut-off score of ≥ 14, and a cut-off score of ≥ 12 to detect dysglycaemia among vulnerable groups across Europe, but that the use of different cut-offs for each subpopulation and country should be considered. In the article, the use of simplified versions of the Finnish Diabetes Risk Score was discussed and suggested to be considered for systematic population screening, as three questions predicted the T2D risk the most, namely, body mass index, use of anti-hypertensive medication, and history of high blood glucose. In addition, age over 45 years was also significantly associated with dysglycaemia (52).

## 4 Discussion

This review contributes to the field of preventive T2D interventions targeting low SES populations. Previous studies have examined barriers and facilitating factors, as well as focus on ethnically diverse populations, in similar contexts (56, 57). Our study enhances understanding of key features in interventions targeting a broad low SES spectrum that are applicable in a high-income country setting, providing a comprehensive view of strategies while also highlighting gaps in coverage.

The results showed, as expected, heterogeneity in study design, content, and outcome measures. We found that most studies were conducted in the United States, and a smaller proportion of studies in Europe. As migration groups, and social welfare and healthcare funding differ from the United States, more studies in European context are encouraged.

Although the interventions in this scoping review were specifically targeting and tailored for populations with low SES, problems with recruitment and retainment were still commonly reported. Previous research on this matter assumed that even when community-engaged approaches are employed, recruiting socially disadvantaged and ethnic minority populations to interventions may take longer than recruiting other populations. Therefore, it was concluded that research institutions need to acknowledge extended time frames when addressing the challenges of research with socially disadvantaged groups (58). To this, we would also add the importance of focusing on the sustainability of participation. According to findings from our study, this could be facilitated by providing flexibility in attendance and format, provision of childcare during sessions, and inviting significant others to sessions. Previous research conducted as part of a thesis, found that involving the people you share a household with is important when making a healthy dietary change; otherwise, the changes are hard to maintain (59).

Only one study tested a digital format of a lifestyle prevention intervention (49). The digitally delivered DPP resulted in weight loss comparable to that observed in studies involving DPP participants from mixed-income populations (60, 61). The limited number of interventions using digital strategies may be attributed to lower levels of digital accessibility among populations with low SES. Computer and online literacy challenges, as well as inconsistent access to computers or the internet, have been reported in a digital health program to prevent diabetes among low-income patients (62). In the study, these challenges led to one-third of the participants among those considered more technically proficient, dropping out. Given that 94% of the population in Sweden has internet access and nearly nine out of 10 use it daily (63), the digital delivery of lifestyle interventions appears feasible in some high-income countries, such as Sweden.

A study reporting a high retention rate in this scoping review was a community-based lifestyle intervention that was both literacy-sensitive and culturally tailored, and included both men and women (44). The intervention resulted in modest but significant weight loss. However, this intervention did not result in any meaningful improvement in physical activity (44). Similarly, the physical activity outcomes in a group-based adaptation of the DPP were disappointing (40). In that study, no supervised physical activities were provided. Instead, participants were encouraged to increase their activity levels, and instructors assisted them in identifying opportunities to incorporate physical activity into their daily routines (40). These findings suggest that the promotion of physical activity in lifestyle interventions may benefit from the inclusion of structured, supervised sessions, as relying solely on encouragement may be insufficient to achieve meaningful behavioral change.

Previous community-based participatory research from the Lindängen initiative for equitable health in Malmö supports gender-specific groups regarding physical activity, as the women who took part in the project wanted a group exclusively for women in order to engage in physical activities (64). At the same time, greater efforts are needed to engage more men in future interventions, as participation rates were lower among men than women in the interventions included in this scoping review. This is particularly important given that T2D affects both genders, with men being slightly more affected (1). Barriers and facilitators to health screening in men have been associated with individual, social, health system, healthcare professional, and screening procedure factors (65). Male peer coaches may serve as positive role models for male participants, and represent a potential strategy for promoting lifestyle changes, as suggested by the study targeting men included in this review (46). For screening interventions, outreach strategies should consider offering services at easily accessible locations and at varied times, as demonstrated by Timm et al. (50), while also taking into account the places where men are typically present in the local context.

In a priority-setting partnership study involving participants with diabetes mellitus and clinicians in the Southwest of Sweden, screening for diabetes detection was identified as one of the top 10 research priorities (66). When we presented preliminary findings from our study to representatives of the local Diabetes Association in the same region, stakeholders were asked which of these interventions they considered most important to initiate in Malmö. Although both lifestyle interventions in various formats, as tested in the included studies, and community-based screening were recognized as highly relevant, the latter was suggested as the preferred starting point. Based on these insights and given that the prevalence of undiagnosed prediabetes and T2D in Malmö is currently unknown, we are now proceeding with an intervention study to address this public health concern, particularly among low SES populations in the city.

### 4.1 Strengths

Three databases were systematically searched, and the search results were independently screened for eligibility by two team members, which is a strength of this study. In addition, the reference lists of the included studies were manually screened, and a fourth database, EMBASE, beyond those specified in the study protocol, was systematically searched to ensure that no relevant studies were missed. Moreover, the results were discussed with members of the local Diabetes Association to enhance the validity of the findings for application in the local context.

### 4.2 Limitations

Despite searching four major databases, there may be gaps in coverage. One limitation of our study is that studies published in journals not indexed in the four selected databases may have been missed. However, the high number of duplicates retrieved (n = 962) suggests good overall coverage for our research question. Furthermore, there is a risk that relevant studies may have been excluded due to the language restriction to English; however, only one study was excluded for this reason during the screening process, and we consider the risk to be low. Grey literature was not searched, as we aimed to map tested and reported interventions. In addition, we aimed for the search strategy to be systematic, transparent, and reproducible, which cannot be ensured when including grey literature (36).

This scoping review aimed to map types of reported interventions for T2D prevention targeting a low SES population. The results will inform future interventions in Malmö, where the prevalence of T2D has doubled between 2011 and 2018. We did not aim to evaluate the effect of the interventions; instead, we based our approach on existing evidence that interventions are effective in preventing T2D and sought to investigate what an adaptation for low SES groups might entail. No risk of bias assessment was conducted, which is consistent with guidance on the conduct of scoping reviews, unless a specific requirement arises due to the nature of the review’s aim, which was not the case in this work (31). We also did not investigate the cost-effectiveness of different types of interventions at this stage. Studies from low-and middle-income countries were not included, as we aimed to focus on studies applicable to high-income countries. However, we may have missed interventions conducted in those countries that could nevertheless be relevant in a high-income country context. Lastly, interventions involving populations under the age of 18 were not included in this scoping review, although such studies could have provided findings relevant to the adult population as well.

## 5 Conclusion

Despite adaptations for the target groups, challenges with both recruitment and completion persisted. Longer timeframes and sustained engagement strategies are necessary to effectively reach and retain groups with low SES in preventive T2D interventions. There is a clear need for interventions in Europe. Cultural adaptations, including flexible formats, facilitating attendance, and the inclusion of significant others in future DPPs, are recommended to improve sustainability for such interventions. Screening interventions reaching different low SES populations should be carried out using the Finnish Diabetes Risk Score questionnaire and be based in the community, in addition to those interventions held at primary healthcare facilities. To increase male participation, future T2D prevention interventions should consider employing male peer coaches and implementing community screenings in settings commonly visited by men.
