# Supplementary material for: Intervention strategies for type 2 diabetes prevention in high-income countries targeting low socioeconomic groups: a scoping review
Source: Front Public Health. 2025 Jul 25;13:1583817. doi: 10.3389/fpubh.2025.1583817 (PMC12331585; doi:10.3389/fpubh.2025.1583817)
Supplement: Supplementary file 1 [file Table_1.docx]

Table 1 Search strategy

| PubMed via NLM January 9 and December 17, 2024 | | |
| --- | --- | --- |
| Diabetes Mellitus | | |
| 1 | Diabetes Mellitus, Type 2[MeSH] OR Prediabetic State[MeSH] OR Diabetes, Gestational[MeSH] OR Glucose Intolerance[MeSH] |  |
| 2 | “type 2 diabetes”[Title/Abstract] OR "diabetes type 2"[Title/Abstract] OR "type 2 diabetes mellitus"[Title/Abstract] OR "diabetes mellitus type 2"[Title/Abstract] OR prediabetes[Title/Abstract] OR "gestational diabetes"[Title/Abstract] OR "impaired glucose tolerance"[Title/Abstract] |  |
| Economic Status | | |
| 3 | 1 OR 2 |  |
| 4 | Low Socioeconomic Status[MeSH] OR Poverty[MeSH] |  |
| 5 | "low socioeconomic status"[Title/Abstract] OR vulnerable[Title/Abstract] OR "low income population"[Title/Abstract] OR "low education"[Title/Abstract] OR "deprived neighbo*"[Title/Abstract] OR "disadvantaged population"[Title/Abstract] OR "disadvantaged area*"[Title/Abstract] |  |
| Prevention | | |
| 6 | 4 OR 5 |  |
| 7 | Primary Prevention[MeSH] OR Life Style[MeSH] |  |
| 8 | prevent*[Title/Abstract] OR lifestyle[Title/Abstract] OR screening[Title/Abstract] OR intervention*[Title/Abstract] OR program*[Title/Abstract] OR strateg*[Title/Abstract] |  |
| 9 | 7 OR 98 |  |
| 10 | 3 AND 6 AND 9 |  |

MeSH – Searches in thesaurus for Medical Subject Headings

Title/abstract - searches in title and abstract

| Cinahl via EBSCO January 12 and December 17, 2024 | | |
| --- | --- | --- |
| Diabetes Mellitus | | |
| 1 | MH "Diabetes Mellitus, Type 2") OR (MH "Diabetes Mellitus, Gestational" OR MH "Prediabetic State" OR MH "Glucose Intolerance" |  |
| 2 | type 2 diabetes OR diabetes type 2 OR prediabetes OR gestational diabetes OR impaired glucose intolerance |  |
| Economic Status | | |
| 3 | 1 OR 2 |  |
| 4 | MH "Low Socioeconomic Status" OR MH "Poverty+" OR MH "Poverty Areas" |  |
| 5 | low socioeconomic status OR vulnerable OR low income population OR low education OR deprived neighbo* OR disadvantaged population OR disadvantaged area |  |
| Prevention | | |
| 6 | 4 OR 5 |  |
| 7 | MH "Life Style+" OR MH "Health Screening+" OR MH "Intervention Trials" |  |
| 8 | prevent* OR lifestyle OR screening OR intervention* OR program* OR strateg* |  |
| 9 | 7 OR 8 |  |
| 10 | 3 AND 6 AND 9 |  |

MH (Medical Headings) – searches in thesaurus for medical headings

| Web of Science via Clarivate January 12 and December 17, 2024 | | |
| --- | --- | --- |
| Diabetes Mellitus | | |
| 1 | TS=("Prediabetic State" OR "Glucose Intolerance" OR "type 2 diabetes" OR "diabetes type 2" OR prediabetes OR "gestational diabetes" OR "impaired glucose intolerance") |  |
| Economic Status | | |
| 2 | TS=("low socioeconomic status" OR vulnerable OR "low income population" OR "low education" OR "deprived neighbo*" OR "disadvantaged population" OR "disadvantaged area") |  |
| Prevention | | |
| 3 | TS=(prevent* OR lifestyle OR screening OR intervention* OR program* OR strateg*) |  |
| 4 | 1 AND 2 AND 3 |  |

TS (Topic) searches in Title, abstract and Keywords

| EMBACE May 29, 2025 | | |
| --- | --- | --- |
| Diabetes Mellitus | | |
| 1 | 'non insulin dependent diabetes mellitus' OR 'impaired glucose tolerance' OR 'gestational diabetes' OR 'glucose intolerance' |  |
| Economic Status | | |
| 2 | (economic status OR 'low socioeconomic status' OR 'poverty' OR 'vulnerable population' OR 'lowest income group' OR low education OR 'disadvantaged population') |  |
| Prevention | | |
| 3 | ('prevention' OR 'primary prevention' OR 'lifestyle modification' OR lifestyle OR intervention OR screening OR 'strategic planning') |  |
| 4 | ([Controlled Clinical Trial]/lim OR [Randomized Controlled Trial]/lim) AND [2020-2025]/py |  |
|  | 1 AND 2 AND 3 AND 4 |  |
