# Supplementary material for: Intervention strategies for type 2 diabetes prevention in high-income countries targeting low socioeconomic groups: a scoping review
Source: Front Public Health. 2025 Jul 25;13:1583817. doi: 10.3389/fpubh.2025.1583817 (PMC12331585; doi:10.3389/fpubh.2025.1583817)
Supplement: Supplementary file 2 [file Table_2.docx]

Table 2 Data extraction Lifestyle interventions

| **Authors, country, year of publication and title** | **Aim of the study** | **Study design** | **Setting** | **Number of participants enrolled** | **Age**  **(mean except noted otherwise)** | **Gender** | **Risk group for T2D** | **SES**  **described as** | **Content** | **Duration of intervention** | **Follow-up time** | **Conclusion/**  **comments** |
| --- | --- | --- | --- | --- | --- | --- | --- | --- | --- | --- | --- | --- |
| Ackermann et al., USA. 2015. A Randomized Comparative Effectiveness Trial for Preventing Type 2 Diabetes | Evaluation of weight loss effectiveness of a YMCA model for the DPP lifestyle intervention. | Randomized comparative effectiveness trial | Delivered by YMCA (an NGO) in an urban setting | N=509  n=257 intervention  n= 252 standard care | 51.0 | W=360  M=149 | Obesity  Prediabetes | Low income | Group-based YMCA adaption of the DPP free of charge; 16 face-to-face, lessons à 60-90 minutes followed by monthly support meetings à 60 minutes.  Goal setting,  self-monitoring, and participant-centered problem solving to achieve modest weight loss through moderate physical activity and lower dietary fat and calorie consumption.  Compared to:  Standard care plus brief lifestyle counseling. | 12 months | 12 months | Completed ≥9 intervention lessons n=103 (40%)  37,4% completed zero intervention lessons    Attending the intervention with >9 visits resulted in 5,3 kg lower body weight at 12 months than they would have if offered brief advice alone. |
| Hays et al., USA. 2016. Effects of a Community-based Lifestyle Intervention on Change in Physical Activity Among Economically Disadvantaged Adults With Prediabetes | Secondary analysis using data from the RAPID study to examine the ability of YDPP as compared to brief counseling to increase PA in a hard-to-reach sample of inner-city adults over 24 months. Additionally, the influence of psychosocial and community-level variables on PA change was examined. | Randomized comparative effectiveness trial | Delivered by YMCA (an NGO) in an urban setting | N=216  n=116 intervention  n=100 control | 53.3 | W= 155 (71,8%) | Obesity  Prediabetes | Low income | **Intervention:**  Brief counseling plus YDPP (based on the DPP lifestyle intervention) with two differences:  1) YDDP was delivered in a group format rather than on an individual basis)  2) YMCA wellness instructors rather than health care professionals delivered program content.  **Control:** briefly delivered (5 minutes) information on the importance of PA on diabetes risk reduction. Participants received a handout describing community resources that supported weight loss and PA programs.  At each follow-up assessment, participants in the control group received repeat diabetes risk testing and brief lifestyle counseling. | 12 months | 24 months | Analysis based on the RAPID participants that wore an accelerometer for 4-7 days and had baseline accelerometer data and accelerometer data for at least one follow-up assessment.  PA outcomes for RAPID participants were disappointing; RAPID participants did lose weight, but they did not significantly change their PA.  Participants might have been more receptive to support provided by peers who share characteristics, values, and lifestyles similar to their own. |
| Carroll et al., USA. 2015. Process Evaluation of Practice-based Diabetes Prevention Programs: What Are the Implementation Challenges? | Feasibility of recruitment and implementation of low-income adults with prediabetes in primary care clinics into diabetes prevention programs. | Pragmatic 2-group pilot comparative effectiveness study of a community-developed Healthy Living program (HLP) and a Diabetes Prevention Program (DPP | Academia, primary care and YMCA (an NGO) in an urban setting | N= 92  n= 42 HLP group  n= 50 DPP group | 48.9 | W=76  M=16 | Obesity  Prediabetes | Low income | **HLP:** 12-week, 24-session program with twice weekly 90 minutes-sessions. Each session consists of 45 minutes of PA and 45 minutes of an interactive, group-based curriculum with nutritional topics.  **DPP:** 3 group and 1 individual meetings per month for 24 weeks. Groups are led by a dietician and/or a PA counselor. Individual meetings (à 30 minutes) alternate between meeting with the nutritionist or the PA counselor every other month. | HLP=12 weeks  DPP=24 weeks | 24 weeks | Completed N= 31 Completed HLP n=13 (31%)  Completed DPP n=18 (36%)  Drop out described as due to life circumstances  job insecurity, competing life issues, logistical barriers.  Lack of standardized procedures to screen for prediabetes  and difficulties retrieving lab results made it difficult to identify eligible patients. |
| Cheyne et al., USA. 2020. Food Bank-Based Diabetes Prevention Intervention to Address Food Security, Dietary Intake, and Physical Activity in a Food-Insecure Cohort at High Risk for Diabetes | To, by a pilot project, assess the effectiveness of a food bank-delivered intervention aimed at improving food security and reducing risk factors for type 2 diabetes among at-risk clients. | Cohort study | Feeding America (a nonprofit organization) and academia  Diverse geo-graphy | N=244 | 48.5 | W=221  M=23 | Self-reported prediabetes  or a high score on CDC´s Prediabetes Risk Test | Low income | Monthly distribution of diabetes-appropriate food packages to participants,  text-based health promotion education (3-5 texts per week) addressing PA and nutrition, administrative and engagement messages, and referrals to health care. | 24-week physical activity module followed by a 24- week nutrition program. | 12 months | Completed postintervention surveys =159    The retention rate was nearly 80% at midpoint (6 months).  Participants had significant improvements in food security status, dietary intake, PA, health status, and depression scores.  Mean BMI did not change.  The intervention demonstrated that food banks can effectively screen clients at high risk for diabetes and improve household food security and other risk factors for diabetes. Food banks may be an important and strategic partner for health care systems or community-based organizations working to prevent diabetes in food-insecure populations. |
| Epel et al., USA.2019. Effects of a Mindfulness-Based Intervention on Distress, Weight Gain, and Glucose Control for Pregnant Low-Income Women: A Quasi-Experimental Trial Using the ORBIT Model | To test the effectiveness of Mindfulness Moms Training (MMT), an intervention in mindfulness training with focus on healthy eating and healthy weight gain during pregnancy | Quasi-experimental trial | Academia | n=110 intervention  n=105 comparison | 27.9 | Women | Obesity  12–19 weeks’ gestation at the start of the intervention | Low income:  <500% of the federal poverty level for a household of 3 which was the median household size in the sample, was used as the upper cut-off, which was close to the median income for San Francisco | 8 weekly 2-h sessions, two “booster” telephone sessions, and one postpartum group session with mothers and babies.  Classes began with mindful movement and a check-in where each person shared their experiences with mindfulness practices in the past week. Didactic discussions then covered (1) stress reduction, focused on acceptance-based coping, awareness of breath, body, thoughts, and emotions; (2) mindful eating, focused on heightening awareness of hunger, fullness, taste experience, and thoughts and emotions leading to reactive and/or automatic eating; and (3) nutrition, focused on optimal foods to eat more of, what to eat less of, reading labels, identifying healthy portion sizes, and introducing both the plate method and food pyramid as resources. Each class ended with a minute mindfulness practice and a review of homework for the upcoming week. | 8 weeks | 8 weeks and for postpartum weight 6-month postpartum | Follow up data available for:  n= 95 in the intervention group and n=90 in the comparison group.  The short-term intervention led to significant improvements in stress and showed promise for preventing glucose intolerance. However, the majority of women gained excessive weight. A longer, more intensive intervention may be needed for this high-risk population. |
| Kim et al., USA 2019. Evaluation of a Digital Diabetes Prevention Program Adapted for Low-Income Patients, 2016-2018 | To examine the effectiveness of a digital DPP adapted for a low-income population. The study was designed to measure participation in the program and its effectiveness in reducing risk for diabetes, compared with a nonparticipating matched group. | Non-randomized matched control trial | Health care  Digital setting | N=227 | 48.2 | W=183 (81.3%) | Obesity  Prediabetes | Enrolled in Medicaid or another safety-net insurance plan | Digital DPP that includes virtual group support, personalized health coaching, weekly lessons, and digital progress tracking tools. The program begins with a 16-week phase, followed by a 36-week maintenance phase. Participants are assigned to small virtual groups with peers and a health coach. Participants complete weekly health education lessons available on the digital platform that can be accessed through the internet or smartphones.  Spanish translation is available and bilingual and -cultural health coaches are present. | 16 weeks followed by a 36- week maintenance phase. | 6 months and 12 months after baseline | More than one-half were highly engaged, completing ≥9 weekly lessons.  In the intervention group mean body weight loss was 4.4 % at the 12 months follow-up.  A digitally delivered intervention, allowing choice of access, can be an effective option in preventing or delaying diabetes by increasing participant engagement. |
| Mayer et al., USA. 2019. Outcomes of a Weight Loss Intervention to Prevent Diabetes Among Low-Income Residents of East Harlem, New York | To examine impact of peer-led diabetes prevention workshops on weight and diabetes risk among a low-income, largely Black and Latino population in East Harlem, New York City. | Cluster blinded randomized trial | Community, health care and academia in an urban setting. | N=402  n=210 intervention  n=192 control (delayed intervention) | 44.5 | W=342 (85%) | Obesity  Prediabetes | Low income  Ethnically diverse population | **Intervention:** The HEED project (help educate to eliminate diabetes) was developed from principles from the DPP and the Stanford Chronic Disease Self-Management Program. Eight 90-minute peer-led workshop sessions conducted in English or Spanish at community sites. Participants could bring a family member, friend, or caregiver to the sessions if they chose. Pairs of peer leaders with similar socioeconomic backgrounds and health problems as the participants led the groups after receiving 4 days of training.  The sessions focused on portion control, simple label reading, managing monthly food budgets, learning to cook healthy food with limited resources, cutting down on unhealthy foods and drinks, reducing sedentary time, environmental factors that promote unhealthy habits (such as limited access to healthy food and poor neighborhood safety/walkability), strategies to deal with stress and emotions that negatively affect health (such as problem solving, contingency management, coping skills, and social support), and incorporating PA into daily life. Participants made action plans, practiced exercise together, brainstormed ideas to address challenges, and contacted each other through a buddy system between meetings. | 8 sessions à 90 minutes  Duration of intervention is not described | 6 months | Completed ≥4 workshops = 64%  Lost to follow up = 25%  At 6 months, the intervention group had lost a significantly greater percentage of their weight and had significantly lower HbA1c rise, decreased risk of diabetes, larger decreases in fat and fiber intake, improved confidence in nutrition label reading, and decrease in sedentary behavior, compared to control group.  Peer led weight loss groups delivered through community-based organizations constitute a promising diabetes prevention strategy among hard-to-reach populations with high rates of diabetes.  Rigorous, community-based screening program were found to be quite feasible and helpful to identify individuals with the highest risk for diabetes and motivate them to action, especially if simple, effective interventions are made available to them. |
| Millard et al., USA. 2011. Pilot of a diabetes primary prevention program in a hard-to-reach, low-income, immigrant Hispanic population. | Evaluation of an exploratory pilot study of an intervention adapted from the Diabetes Empowerment Education Program to reduce diabetes risk factors in a South Texas colonia population with a high rate of overweight and obesity. | Pre-post-design with a comparison group | Academia in a rural setting | N=81  n=38 intervention group  n=43 comparison group | 36 years inter-vention group  35 years com-parison group | Data available for them who attended at least half of the meetings:  W=38  (100%) intervention group  W=41 (95%) comparison group | A general population with an extra-ordinarily high mean BMI  Ethnic group with a genetic predisposition prone to develop T2D | Low income | Community-based participatory approach and adaptation of the Diabetes Empowerment Education Program. Childcare was provided at the same locations as the meetings.  Content in the meetings:  1. Understand the human body  2. Understanding chronic diseases and chronic disease risk factors  3. Monitoring your body  4. Get up and move (PA)  5. Controlling chronic disease through nutrition  6. Diabetes complications: identification and prevention  7. Living with chronic disease, mobilizing family and friends  8. Final celebratory meal (meal preparation, family was invited)  Walking groups were formed by the participants and the promoters led the design of many of the culturally appropriate aspects of the project, and the researchers consistently supported their initiatives. | 8 weekly meetings | At the end of the weekly meetings | In the intervention group, 79.2% attended at least half of the meetings.  The intervention resulted in an average change in BMI that was small but statistically significant.  The study shows that low-income, rural Mexican American families will take ownership of a program that is participatory and tailored to their culture and economic situation. |
| Ockene et al., USA. 2012. Outcomes of a Latino community-based intervention for the prevention of diabetes: the Lawrence Latino Diabetes Prevention Project | To test the effectiveness of a community-based, literacy-sensitive, and culturally tailored lifestyle intervention on weight-loss and diabetes risk-reduction among low-income, Spanish-speaking Latinos at increased diabetes risk | Two arm- randomized controlled study | Community-academic partnership  in an urban setting | N=312  n= 162 intervention  n= 150 control | 52 | W=74%  n=117 women in inter-vention group  n= 115 in control group | Obesity  Ethnic group with a genetic predisposition prone to develop T2D  Calculated risk of 30% or higher to develop T2D over the succeeding 7.5 years | Low income, 60% Latino city | The community-based, culturally tailored, literacy-sensitive lifestyle intervention was delivered by Spanish-speaking community individuals, and was based on, but modified, the DPP and included participation in 3 individual and 13 group sessions over a 12-month period à 30-90 minutes. Makeup sessions were offered when participants missed group sessions.  A healthy meal was served at all sessions and preparation methods were discussed. Significant others were invited to attend each group session.  A picture-based food guide that classified foods into 3 colors (green, yellow or red) with regard to glycemic index, sodium and saturated fat was used during a supermarket tour to assist participants. | 12 months | 12 months | A community-academic partnership enabled the successful recruitment, intervention, and assessment of Latinos at risk of diabetes with a one-year study retention rate of 93%.  The lifestyle intervention tested in the study led to a modest but significant and clinically meaningful weight loss among participants in the intervention group compared to control group. Weight loss was associated with a significant reduction in insulin resistance and HbA1c. No meaningful improvement in PA was achieved, and was discussed may be related to lack of emphasis on this aspect of the intervention and that the neighbourhood was not conducive to PA. |
| Philis-Tsimikas et al., USA. 2014. Dulce Mothers: an intervention to reduce diabetes and cardiovascular risk in Latinas after gestational diabetes | To examine effectiveness of a DPP translation in low low-income Latinas with a history of GD | Single-group pre-post design | Community clinic in a county setting | N=84 | 31.9 | Women | Ethnic group with a genetic predisposition prone to develop T2D  GD in the past 3 years | Low-income  Minority population | An 8-week 2-hour peer-educator-led group intervention, with tailoring for Latino culture and recent motherhood. The schedule was chosen, instead of 16 1-h sessions as in the DPP, to reduce attendance barriers.  Consistent with DPP adaptions the curriculum is grounded in a social cognitive theory and focuses om developing health-related knowledge, behavioral skills such as goal setting and self-monitoring, building resources (e.g. self-efficacy and support) for enacting health protective behaviors, and learning via role modeling (i.e. form peer educators and other group members). All core topics in DPP were delivered in Spanish and presented in an abbreviated manner and discussion of the topics specific to Latina mothers including breastfeeding, childhood obesity, modeling healthy behaviors to family members, and nonfood rewards for children. Furthermore, discussions were conducted about culturally driven beliefs about diabetes and income-related barriers to healthy lifestyles. | 8 weeks | 6 months | The enrollment-rate was 53%.  Reasons for attrition included inability to reach/disconnected number, lack of transportation, or return to Mexico. Only two women expressed lack of interest.  A peer-led, culturally appropriate DPP translation was effective in improving lifestyle changes and some indicators of cardiovascular and diabetes risk in Latinas with GD. |
| Rautio et al., Finland. 2012. Participation, socioeconomic status and group or individual counselling intervention in individuals at high risk for type 2 diabetes: one-year follow-up study of the FIN-D2D-project. | To describe socioeconomic characteristics of participants and their effect on uptake and completion of the implementation project (FIN-D2D) for the National Type 2 Diabetes Prevention Programme. Furthermore, to assess the effectiveness of individual vs. group intervention during one-year follow-up. | Non-randomized implementation project | Primary care, real world setting | N=8584 | W=51.2  M=52.6 | W=5764 M=2820 | High-risk cohort  according to FINDRISC | Education and occupation, analyzed after intervention | Participants willing to adopt lifestyle changes, selected the type of intervention (individual or/and group counselling) based on their own needs together with health professionals.  Both types of interventions were based on empowerment and were tailor-made for each individual. A multi-professional team planned and agreed the agenda and methods for the sessions together with participants.  Group counselling for 8-10 individuals four times at intervals of 1-2 weeks, with a fifth follow-up session one month after the last session, led by nurses. Mainly exercise groups and about weight maintenance.  Individual visits included e.g. information on weight reduction, a healthy diet, PA and smoking. | Not specified | 1 year | 69.8% of the women participated in some of the intervention modalities offered.  Low education and not working were related to active participation in the intervention in men.  88.2% of men and 76.1% of women selected the individual instead of group intervention.  The effectiveness of individual vs. group interventions did not differ, except for minor changes in systolic blood pressure in women and glucose tolerance in men. |
| Ritchie et al., USA. 2023. Reducing Maternal Obesity and Diabetes Risks Prior to Conception with the National Diabetes Prevention Program | The aim was to provide initial evidence of the National Diabetes Prevention Program´s (NDPP) effects on maternal-child outcomes in diverse, low-income women and their offspring | Observational study | Healthcare | n= 32  in NDPP group    compared to  n= 26 receiving usual care | 30.2 in NDPP  26.9 in usual care control | Women | BMI >25 kg/ m²  or >23 kg/ m² if Asian ancestry  in addition to having prediabetes, past GD, or a positive score on a risk questionnaire | Participants were part of a safety-net healthcare system  Diverse and low-income population | The NDPP is available in all US States and targets 5% weight loss and ≥150 min of PA per week for adults with diabetes risks.  Participants were identified primarily from provider referrals and enrolled into group classes. Each class included 22-24 hourlong sessions over one year. Sessions were led by bilingual (English and Spanish) lifestyle coaches. Coaches also outreached to participants between sessions to provide individual support and offer make-ups as needed. | 1 year | Related to time for con-ception, pregnancy and giving birth | Women achieved nearly 3% weight loss in the NDPP, and usual care resulted in 3% weight gain over 1.5 years.  16% of women still developed GD after participating in the NDPP, but almost all cases manifested later in pregnancy. In contrast, all 23% of GD cases that occurred in usual care had also screened positive for hyperglycemia in early pregnancy.  Results provide preliminary evidence that the NDPP may support a reduction in peri-conceptual obesity/diabetes risks among diverse and low-income women |
| Thomas et al, France. 2022. Adapted educational health program among deprived subjects with prediabetes | To assess the feasibility and benefit if a health educational program in global metabolic status in prediabetic deprived subjects | Non-randomized controlled study | Academia and health care  Urban setting | n= 133 intervention  n=415  control  n=145  abandoned group | 55.6 intervention group  51.3 control group  49.5 abandon group | W=41 (31%)  M=92  intervention group  W=140 (33.7%) M=275  control group  W=46 (31.7%)  M=99  Abandon group | Prediabetes  as defined by ADA | Socioeconomic deprivation assessed by the validated EPICES score (Evaluation de la Précarité et des Inégalités de santé) | 4 workshops à 90 minutes (2 nutrition and 2 PA workshops). The scheduling of workshops considered the subject´s availability. When the subjects had participated in the 4 workshops, one year after the first visit, they were offered a new health-check-up.  Dieticians and physical education experts led the workshops and were certified for educational group-therapy. | Flexible and consi-dering the subject´s availa-bility, but planned for a year | 21 months in average | The study confirmed that a minimal suited and supervised educational program among deprived subjects with prediabetes tended to limit fasting glucose increment, improve metabolic status as well as encourage a lifestyle aimed to limit cardiovascular and diabetes risk factors. The study highlights difficulties to convince subjects with socioeconomically deprived profile to participate in such workshops since only 20% participated to the entire study. |
| Walker et al., USA. 2018. Power Up for Health: Pilot Study Outcomes of a Diabetes Prevention Program for Men from Disadvantaged Neighborhoods | To better engage men of colour with prediabetes from disadvantaged neighbourhoods of New York City. | Quasi-experimental intervention design | Academia  Urban setting | N=29 | 49.9 | Men | Obesity  Prediabetes | Low income | The Power Up for Health program included the 16-weekly, 1 h core sessions of the NDPP, with the curriculum adapted to better engage men from disadvantaged, urban neighbourhoods. The program was led by male lifestyle health coaches. One coach was Spanish speaking. The program was offered at five different recreation sites. If a participant missed to attend a weekly session, the coaches proactively offered a telephone make-up of that session to each participant to be done after they received the session materials by email or by mail. | 16 weeks | 16 weeks | Participants engaged in ≥4 sessions n=25  The average weight loss was 5.6%.  The average number of attended sessions were 11.6.  Coaches, participants, and the Advisory Panel suggested addition of group physical activity at the time of each session to improve the program.  Authors suggested to improve program by offering group sessions by telephone to decrease weekly attendance burden for men with prediabetes from disadvantaged neighbourhoods. |

ADA: American Diabetes Association
BMI: Body mass index
CDC: Centers for Disease Control and Prevention
DPP: Diabetes prevention program
GD: Gestational diabetes
NDPP: National Diabetes Prevention Program
OGTT: Oral glucose tolerance test
PA: Physical activity
WHO: World health organization
