# Supplementary material for: Intervention strategies for type 2 diabetes prevention in high-income countries targeting low socioeconomic groups: a scoping review
Source: Front Public Health. 2025 Jul 25;13:1583817. doi: 10.3389/fpubh.2025.1583817 (PMC12331585; doi:10.3389/fpubh.2025.1583817)
Supplement: Supplementary file 3 [file Table_3.docx]

Table 3 Data extraction Screening interventions

| **Authors, country, year of publication and title** | **Aim of the study** | **Study design** | **Setting** | **Number of participants enrolled** | **Age**  **(mean except noted otherwise)** | **Gender** | **Risk group for T2D** | **SES**  **described as** | **Content** | **Duration of intervention** | **Follow-up time** | **Conclusion/**  **comments** |
| --- | --- | --- | --- | --- | --- | --- | --- | --- | --- | --- | --- | --- |
| Goyder et al., England. 2008. Evaluating the impact of a national pilot screening programme for type 2 diabetes in deprived areas of England | To evaluate the impact of screening on the prevalence of type 2 diabetes. | Prospective study | Primary care  In an urban setting | N= 41 418 invited  n=25 356 screened | Data available on screening-detected diabetes  N= 12145  <40 y: n=618  40-59 y n=6840  60-69 y n=3958  80+ y 651  Missing: n=78 | Data available on screening-detected diabetes N=12 145  W=6503  M=5589  Missing information=53 | Population expected to have a high prevalence of undiagnosed diabetes | Socio-economically deprived  Ethnically diverse | The pilot practices were asked to use their clinical information systems to identify patients >40 years with a BMI >25 and offer a random capillary blood glucose test. A result of >6 mmol/l was suggested to indicate eligibility to for diagnostic testing (OGTT or fasting blood glucose).  Each area had a facilitator who offered practical support to the practices and the practices were reimbursed for the costs of additional screening activity. | 2 years. 2003 and 2005 | N/A | 1 in 70 people screened was given a new diagnosis of diabetes.  The ‘real world’ nature of the program and dependence on routine data collection systems made results more difficult to interpret but also enabled problems with implementation, not evident from previous research, to be identified. It is likely that the low diagnostic yield was largely due to a high level of ad hoc screening activity outside the pilot protocol and inadequate access to diagnostic testing after a positive screening test. Implementation of screening for diabetes in primary care should not be undertaken without robust assessment of the resources required for diagnostic testing and follow-up and adequate clinical audit. |
| Mavrogianni et al., Belgium, Finland, Greece, Spain, Bulgaria, Hungary. 2019. Evaluation of the Finnish Diabetes Risk Score as a screening tool for undiagnosed type 2 diabetes and dysglycaemia among early middle-aged adults in a large-scale European cohort. The Feel4Diabetes-study | To assess the diagnostic accuracy of the FINDRISC in detecting undiagnosed T2D and dysglycaemia among early middle-aged adults from vulnerable groups in a large-scale European cohort, and to examine any potential  differences in FINDRISC performance among LMICs and vulnerable groups in HICs and HICs under austerity measures.  A secondary aim was to examine which FINDRISC components were most strongly associated with these outcomes  to further support the development and use of simplified versions. | Cohort study | School-/community- based  10 partners within academia, a research institute, an advocacy group and an enterprise | N=2116 | 41.1 | W=1423 (67.3%) | Based on self-reported FINDRISC-score | In Bulgaria and Hungary all families were  considered vulnerable and eligible to participate in the study,  while in Belgium, Finland, Greece and Spain, families from municipalities with the lowest educational level or  the highest unemploy-ment rate were included as vulnerable  groups. | All parents were requested to complete the FINDRISC questionnaire.  Fasting blood glucose and serum total and high-density lipoprotein, cholesterol and triglyceride levels were analyzed.  Participants without previously diagnosed diabetes were classified according to the WHO criteria as:  Normal (FPG <6.1 mmol/L), prediabetes (FPG 6.1-6.9 mmol/L) and T2D (FPG ≥7.0 mmol/L). | Measurements were conducted between April and September 2016 | N/A | The data further supports the use of the FINDRISC as a useful self-administrated tool and the first step in screening large multinational populations and identifying individuals primarily with undiagnosed T2D but also with dys-glycaemia. In the study population, a cut-off ≥14 found to be the most suitable value for identifying undiagnosed T2DM, while a cut-off ≥12  proved to be optimal for the detection of dys-glycaemia. The different cut-offs identified for each country category could also be taken into consideration in the practical application, while the use of any simplified version of the FINDRISC could also be considered for systematic population screening. |
| Timm et al., Sweden. 2020. Early detection of type 2 diabetes in socioeconomically disadvantaged areas in Stockholm - comparing reach of community and facility-based screening | To determine the difference in population reach and participant characteristics between community- and facility-based screening for detection of T2D and persons at high risk of developing diabetes. | Cross sectional study | Health care, citizen service offices, local organizations and academia  Suburban setting | N=2564  Community screening n= 1827  Facility screening n=737 | Median age was 50 years of age  (48 in the com-munity- and 56 in the facility- screened group) | W=1118 (44%) | FINDRISC- score >12 /25 was defined as having high risk and referral for HbA1c-testing | Suburbs characterized by low income, low education and high unemployment rates  High proportion of migrants | Screening via FINDRISC, and if $\geq$12, offering a point of care HbA1c, and if >38 mmol/mol referral to primary health care center for further examinations.  Most individuals were screened at shopping malls. Other places targeted were local organizations, associations, women´s centers and swimming facilities. The screenings were conducted at different times throughout the day and the entire week including weekends.  The community-based screening was compared to facility-based screening. | March 2017 – June 2018 | N/A | In the community-based screening 43% had high risk of T2D according to FINDRISC. Community-based screening and facility-based screening were found to be accessed by different population groups with some overlap. Thus, both screening  methods are needed to reach persons at high risk of  developing T2D. The study found that it is particularly important to implement screening in socioeconomically disadvantaged areas where the prevalence  of diabetes and diabetes risk is higher than in the  general population. Community screening reached more hard-to-reach groups with unfavorable risk profiles, making it a critical strategy for T2D  prevention. Facility-based screening reached more people born in Sweden and other European countries. |
