# Supplementary material for: Intervention strategies for type 2 diabetes prevention in high-income countries targeting low socioeconomic groups: a scoping review
Source: Front Public Health. 2025 Jul 25;13:1583817. doi: 10.3389/fpubh.2025.1583817 (PMC12331585; doi:10.3389/fpubh.2025.1583817)
Supplement: Supplementary file 4 [file Table_4.docx]

Table 4 Outcome measures

| **Focus of study** | **Authors** | **Primary outcome measure** | **Secondary outcome measures** |
| --- | --- | --- | --- |
| Risk for or history of GD | Epel, et al., 2019 | Gestational weight gain | **Psychosocial Distress** measured using standardized scales: Global perceived stress using Cohen’s Perceived Stress Scale Depressive symptoms using the Patient Health Questionnaire (PHQ-9) Pregnancy-related anxiety (using the Pregnancy-Related Anxiety Scale)  **Mindfulness Outcomes** Acceptance of negative experiences (using the Acceptance and Action Questionnaire-II)  **Trait mindfulness   Eating Behaviors** measured using standardized scales Emotional eating (using the Dutch Eating Behavior Questionnaire) External eating behavior (using the Dutch Eating Behavior Questionnaire Food addiction (using the Yale Food Addiction Scale)  **Metabolic Health** Oral Glucose Tolerance Test (OGTT) glucose levels Glucose levels 1-hour post-test were abstracted from medical records for participants who completed the test between 24 and 28 weeks' gestation  **Impaired glucose tolerance** Categorically defined as a glucose level above 130 mg/dL after the 1-hour OGTT  **Physical Activity** Assessed at baseline and post-intervention using the Stanford Brief Activity Survey  **Six-Month Postpartum Weight Retention** |
| Risk for or history of GD | Philis-Tsimikas, et al., 2014 | HbA1c | **Lipids**: Total cholesterol, high-density lipoprotein cholesterol (HDL-C), low-density lipoprotein cholesterol (LDL-C), and triglycerides **Blood pressure**: Systolic blood pressure (SBP) and diastolic blood pressure (DBP) **Body Mass Index (BMI) Self-reported outcomes:** Physical activity, using the Rapid Assessment of Physical Activity (RAPA), including aerobic activity and flexibility/strength training Dietary fat intake (% of total calories) Overall perceived health Diabetes-specific cultural beliefs |
| Risk for or history of GD | Ritchie, et al., 2023 | BMI change from baseline to conception Obesity at conception HbA1C change from baseline to early pregnancy (%)  Diagnosed with GDM by late pregnancy Gestational weight gain at 37 + weeks Excess gestational weight gain at 37 + weeks (Offspring outcomes not presented in this scoping review) |  |
| Screening intervention | Goyder, et al., 2008 | Number of newly diagnosed cases within the screening programme  Diagnostic yield  The number needed to screen to diagnose one additional case  Change in overall diabetes prevalence over a 2-year period | Outcomes related to the screening process and implementation, which affected the diagnostic yield |
| Screening intervention | Mavrogianni, et al., 2018 | FINDRISC's ability to detect undiagnosed T2DM and dysglycaemia among early middle-aged adults from vulnerable groups in a large European cohort  Prevalence |  |
| Screening intervention | Timm, et al., 2020 | FINDRISC questionnaire  The reach of the different screening methods (community- and facility-based) to different population segments in socioeconomically disadvantaged areas |  |
| Adapted DPP | Ackerman, et al., 2015 | Change in body weight over 12 months | The percentage of participants who reached weight loss goals of 5% or greater  Changes in blood pressure  Changes in total cholesterol  Changes in high-density lipoprotein (HDL)-cholesterol  Changes in HbA1c  Survey data collected at each visit was used to assess potential unanticipated harms and secondary clinical outcomes such as cardiovascular events |
| Adapted DPP / community-developed Healthy living program | Carroll, et al., 2015 | The feasibility of recruitment and randomization  The numbers of potentially eligible participants, those enrolled and randomized, and reasons for ineligibility, barriers to randomization and enrollment, and nonparticipation  Participant flow through the study (as shown in a CONSORT diagram)  Retention in the program  Reasons for non-retention  Participant satisfaction and suggestions for improvement, collected via a post-study survey |  |
| YMCA adaptation of the DPP or YDPP | Hays, et al., 2016 | Changes in physical activity calculated from accelerometer counts | Behavioral mediators were assessed through survey data, including readiness to change, self-efficacy, social support, diabetes risk perception, self-reported physical activity, and perceived health status |
| Digital DPP | Kim, et al., 2019 | Change in weight   Percentage who lost more than 5% of their baseline weight and change in BMI  HbA1c  Engagement, measured as the number if completed lessons (0-16) |  |
| Partnership-developed diabetes prevention program based on principles derived from DPP and the Stanford Chronic Disease Self-Management Program | Mayer, et al., 2019 | The proportion of participants who achieved 5% weight loss  Percentage weight loss  Change in the probability of developing diabetes over the next 7.5 years according to the San Antonio Diabetes Prediction Model | Change in weight, BMI, HbA1c, diet (fruit, vegetable, fat, and fiber intake), nutrition label reading frequency and confidence and sedentary (screen) time |
| Adapted Diabetes Empowerment Education Program (DEEP) | Millard, et al., 2010 | Change in BMI |  |
| Adapted DPP | Ockene, et al., 2012 | Weight loss  HbA1c | Fasting lipids (total cholesterol, HDL cholesterol, triglycerides, and calculated LDL cholesterol)  Fasting glucose  Fasting insulin concentrations  Blood pressure  Dietary assessment  Physical activity measurements  Quality of life scores (Short Form-12 (SF-12)  Depression scores using Center for Epidemiological Studies-Depression Scale (CES-D) |
| Adapted NDPP | Walker, et al., 2018 | Weight loss | Changes in assessments of healthy eating and physical activity using items from the Summary of Diabetes Self-Care Activities (SDSCA)  Self-reported health status using a one-item question from the National Health Interview Survey  Screen for depressive symptoms using the 2-item Patient Health Questionnaire-2 (PHQ-2)  Program attendance, including in-person attendance and telephone make-up sessions |
| Monthly distribution of diabetes-appropriate food packages, text-based health promotion education and referrals to healthcare | Cheyne, et al., 2020 | Changes in household food security  Physical activity  Consumption of healthy foods (particularly fruits and vegetables)  Weight and BMI |  |
| Finnish National T2DPP | Rautio, et al., 2012 | BMI  Waist circumference  Systolic and diastolic blood pressure  Levels of total cholesterol, HDL cholesterol, and triglycerides  LDL cholesterol concentration  Fatal cardiovascular risk for 10 years (estimated using the SCORE risk estimation system)  Glucose Tolerance:  Fasting glucose levels  2-hour glucose levels from an oral glucose tolerance test (OGTT)  Change in glucose tolerance status (classified as deteriorated, no change, or improved)  The incidence of T2D |  |
| Adapted educational health program | Thomas, et al., 2022 | Fasting blood glucose variation Systolic blood pressure variation Weight BMI Waist circumference variation Quality of life using the SF36 questionnaire score | Self-questionnaire regarding their satisfaction regarding the lifestyle counselling |
