# Supplementary material for: Intervention strategies for type 2 diabetes prevention in high-income countries targeting low socioeconomic groups: a scoping review
Source: Front Public Health. 2025 Jul 25;13:1583817. doi: 10.3389/fpubh.2025.1583817 (PMC12331585; doi:10.3389/fpubh.2025.1583817)
Supplement: Supplementary file 5 [file Table_5.docx]

Table 5 SES Indicators used in the included studies

| **SES indicator** | **Definition/Cut-off** | N**umber of studies of 17 studies** |
| --- | --- | --- |
| Education | <High school/High school diploma/More than high school (Carroll, et al., 2015)  <High school or General education diploma/Completed high school or General education diploma/Some college, but no degree/Completed 2-year degree/Completed 4-year degree or higher (Cheyne, et al., 2020)  <12th grade, high school graduate or General education diploma/any college or vocational training/college graduate or higher (Epel., et al, 2019)  Some high school/High school diploma or General education diploma/Some college or college graduate (Kim, et al. 2019)  <12 years/>12 years (Mavrogianni, et al., 2019)  Never attended school or only kindergarten/Elementary/Middle school/High school/Some college or technical school/College graduate/Refused to answer (Millard, et al., 2011)  Less than high school education (Mayer, et al., 2019; Ockene, et al., 2012)  Educational attainment <High school ≥Some high school (Philis-Tsimikas, et al., 2014)  Low (primary, basic, middle school, vocational school, or upper secondary) or high (college, polytechnic, academic degree) (Rautio, et al., 2012) | 9 |
| Occupation/Employment | Not employed/Retired, disabled, homemaker, stay at home-parent, or student/Employed in temporary or part-time job/Employed full-time/Other (Cheyne, et al., 2020)  Unemployed/Employed part-time/Employed full-time (Kim, et al., 2019)  Unemplyed/Stay at home parent/Work part-time/Other (Mavrogianni, et al., 2019)  Salary/hourly employee/Self-employed/Unemployed for more than 1 year/Unemployed for less than 1 year/Homemaker/Student/Retired/Unable to work (Millard, et al., 2011)  Employed, number/percentage (Ockene, et al., 2012)  Employed outside of the home: Yes/No (Philis-Tsimikas, et al., 2014)  Preset options: (1) agriculture, animal husbandry or forestry, (2) factory work, mining, construction or corresponding work, (3) clerical, service or intellectual work, (4) studying, (5) housewife/husband, (6) retired and (7) unemployed. Occupation was also categorized into two classes: classes 1–4 were combined to indicate being actively engaged in work or study, while classes 5–7 represented not working or studying (Rautio et al., 2012) | 7 |
| Income | $0-$999/$10 000–$19999/$20 000 –$29 999/$30 000–$39 999/$40 000 or more/Did not wish to answer (Carroll, et al., 2015)  <$15 000/year (Meyer, et al., 2019) | 2 |
| Household income/year | <$20 000/$20 000–39 999/$40 000–59 999/$60 000–79 999 (Cheyne, et al., 2020)  <$10,000/10,000–$14,999/$15,000–$24,999/$25,000–$34,999/$35,000–$49,999($50,000–$74,999 (Hays, et al., 2017)  <$10 000/10 000–24 999/>25 000 (Ockene, et al., 2015)  <$12 000/$12 000–$24 000/>$24 000 (Philis-Tsimikas, et al., 2014) | 4 |
| Home ownership | Rent/Own/Other (Cheyne, et al., 2020) | 1 |
| Race/Ethnicity/Migrant history | Asian/Black or African American/Hispanic or Latino/Other (Cheyne, et al., 2020)  Non-Hispanic white/Hispanic or Latino/African American/Other or preferred not to answer (Carroll., 2015; Kim, et al., 2019; Ockene, et al., 2015; Walker, et al., 2018)  White/African American/Latino/Other or multiracial (Epel, et al., 2019)  White/Black/South Asian/Other/Missing (Goyder, et al., 2008)  Non-Hispanic Black/Hispanic/Non-Hispanic white/Other (Mayer, et al., 2019) Foreign-born (Mayer, et al., 2019)  People began their education in Mexico/United States/Other/Refused to answer/No response (Millard, et al., 2011)  Nativity: Mexico/US/Other (Philis-Tsimikas, et al., 2014)  Latinx/Non-Latinx Black/Non Latinx white/Other (Ritchie, et al., 2023) | 11 |
| Marital status | Single, separated or divorced/married or in a committed relationship (Epel, et al., 2019)  Married pr cohabitating/Separated/Never married (Philis-Tsimikas, et al., 2014)  Married or living together, number/percentage (Ockene, et al., 2012) | 3 |
| Deprivation Indices | The Care Need Index (CNI)  CNI is a social deprivation index that uses socio-demographic variables to construct a composite score used as an indicator to assess need of care in a population. The variables used in the construction of the CNI score include: Number of persons aged over 65 living alone; born abroad (Eastern Europe, Asia, Africa and South America); unemployed (aged 16–64); single parents with children 17 years or younger; persons who moved into the area (1 year or older); persons with low education (aged 25–64); children younger than five years. In the present study, the CNI was higher in these areas compared to more affluent suburbs in Sweden (Timm, et al., 2020)  Evaluation de la Précarité et des Inégalités de Santé (EPICES score). The EPICES score has been validated and ranges from 0 to 100: the higher the score, the higher the level of socioeconomic deprivation. No fixed threshold was recognized to determine deprivation. However, subjects with a score greater than 30, corresponding to the median score in the population of 693 subjects, were considered socioeconomically deprived for the study (Thomas, et al., 2022). | 2 |
| Food security | The 10-item U.S. Adult Food Security Module 1 (Epel, et al., 2019)  The USDA Economic Research Service´s 6 -item screener (Cheyne, et al., 2020)  Food insecure – assessment not defined (Mayer, et al., 2019) | 3 |
| Participation in the Supplemental Nutrition Assistance Program (SNAP) | Yes/No (Cheyne, et al., 2020) | 1 |
| Insurance Status/Type | Public, Private, None (Carroll, et al., 2015)  Medicaid or Medi-Cal/Other program for uninsured (Kim, et al., 2019)  Uninsured/Medicaid/Medicare/Commercial  (Mayer, et al., 2019)  Proportion with no health insurance (Philis-Tsimikas, et al., 2014)  Medicaid/Uninsured/Private (Ritchie, et al., 2023) | 5 |
| Community-level variables | Food Desert Locator Walk Score  Uniform Crime Report Median Household Income and Use of Public Transportation  Vegetation Index (Hays et al., 2016) | 1 |
| Descriptive | Low-socioeconomic areas or areas with characteristics like low educational level and high unemployment rate as retrieved from official  resources and authorities (Mavrogianni, et al., 2019)  High levels of socio-economic deprivation and multi-ethnic populations (Goyder, et al., 2008)  The 2008 household income was $33 684 in Lawrence, compared with $65401 for Massachusetts overall (Ockene, et al., 2012)  “Disadvantaged” was used to designate communities characterized by high poverty rates and low resources (Walker, et al., 2018) | 4 |
