# Supplementary material for: Intervention strategies for type 2 diabetes prevention in high-income countries targeting low socioeconomic groups: a scoping review
Source: Front Public Health. 2025 Jul 25;13:1583817. doi: 10.3389/fpubh.2025.1583817 (PMC12331585; doi:10.3389/fpubh.2025.1583817)
Supplement: Supplementary file 6 [file Table_6.docx]

Table 6 Key intervention features

| **Key intervention features** | **Description of content**  **(number of studies of 17 studies)** |
| --- | --- |
| Population engagement | Community-based participatory approach (3) |
|  | Scheduling sessions in collaboration with the participant (1) |
|  | Flexible attendance (3) |
|  | Providing a choice between individual or group sessions (2) |
|  | Practice-based activities included, e.g. cooking, physical activity, mindful movement (5) |
| Delivery format | Group based sessions (13) |
|  | Digital sessions (1) |
|  | Outreach delivery of diabetes-appropriate food-packages (1) |
|  | Text-based messages or chat (2) |
|  | “Booster” telephone sessions between sessions (1) |
| Involving significant others | Celebratory meal together with family (1) |
|  | Participants could bring a family member, friend, or caregiver to the sessions if they chose (3) |
|  | Providing childcare during sessions (2) |
| Cultural and Language Adaptation | Bilingual or in a preferred language (7) |
|  | Coaches with similar background (1) |
|  | Discussions about cultural beliefs regarding T2D (1) |
|  | Inclusion based on a specific ethnicity (7) |
| Gender focus | Inclusion based on being a woman (3) |
|  | Inclusion based on being a man (1) |
| Screening for persons at risk for T2D | Using the FINDRISC questionnaire (2) |
|  | Through healthcare facilities (2) |
|  | Community-based (2) |
|  | Outreach through schools (1) |
